# Checklists as a central part of surgical safety culture

**DOI:** 10.1590/1516-3180.2022.140404052022

**Published:** 2022-08-08

**Affiliations:** I MD, PhD. Coordinator of the Surgical Quality and Safety Unit, Cardiac Surgery Division, Instituto do Coracao, Hospital das Clinicas HCFMUSP, Faculdade de Medicina, Universidade de Sao Paulo, Sao Paulo, SP, BR.; II MD, PhD. Titular Professor of the Thoracic Surgery Program, Instituto do Coracao, Hospital das Clinicas HCFMUSP, Faculdade de Medicina da Universidade de Sao Paulo, Sao Paulo, SP, BR; and Director of the Scientific Department, Associação Paulista de Medicina, São Paulo (SP), Brazil.; Associação Paulista de Medicina, Scientific Department, São Paulo, SP, Brazil

The number of surgical procedures performed is increasing worldwide. In 2004, the number of major operations performed reached 281 million, i.e. approximately one operation per year for every 25 individuals.^1^ However, the numbers of complications among surgical patients has also been increasing and this has become the greatest cause of death and disability worldwide.^2^ A systematic review has demonstrated that one in every 150 hospitalized patients dies as a consequence of complications related to an adverse event and that almost two thirds of these deaths are associated with surgical treatment.^3^ Half of these adverse events are considered to be avoidable.^4^

In the 1970s, following a series of air accidents, analysis on these events demonstrated that a combination of stress, fatigue, lack of communication and avoidable errors caused up to 80% of them.^5^ Through use of safety checklists and continuous training for crews, the incidence of air accidents has continually fallen since then, despite significant increases in the volume of air traffic. These checklists are now used routinely in aviation and other high-complexity industries.^6^ Use of checklists offers a singular opportunity to correct any problems before proceeding and provides awareness of situations that are still to come.

Faced with such evidence regarding patient safety, in 2002 the World Health Organization (WHO) adopted resolution 5518 (WHA 55.18), which called on its member countries to strengthen the care taken regarding safety, and demanded standardization of norms in order to construct a culture of surgical safety. Soon afterwards, in May 2004, it launched the campaign “WHO Patient Safety”, in which leaders of prominent healthcare institutions, political representatives and patient groups around the world came together with the aim of reducing the numbers of adverse events caused by lack of care for patients.

At the first meeting, in January 2007, difficulties in improving surgical safety were identified and reviewed. The concept of surgery was defined as: “Any procedure that takes place in an operating theater, involving incision, excision, manipulation or suturing of tissues, which would normally require regional anesthesia, general anesthesia or deep sedation in order to control pain”. It was recognized that surgical safety is multifactorial and requires reliable implementation of a variety of measures that are needed for attending to patients, not only by the surgeon but also by the entire team of professionals who work together for patients’ benefit.

It has been observed that reliability in various areas of medicine can be improved through identifying the care to be provided and standardizing the routines. This can be done through using tools such as safety checklists. The WHO checklist consists of a simple instrument that is divided into three parts or sections. The first part is applied before induction of anesthesia; the second, before the incision in the skin is made; and the third, before the patient leaves the operating theater. These checks make us feel that we are within a system, thus improving communication and preparing us for the unexpected.

The checklist is composed of items such as confirmation of the patient’s identity, the location of the surgical site and the type of procedure to be performed; and items relating to checking ventilation and monitoring. The list includes data on the patient’s allergies, airways and risk of bleeding, and for anticipating critical events. Within this process, there is a “time out” moment^7^ immediately before the skin incision is made, at which all members of the team give verbal confirmation of the patient’s identity, the location of the surgical site and the procedure to be performed. This pause prepares us in the same way as if we were airplane pilots about to take off, with a focus on items that could cause danger.

The WHO checklist was tested in eight countries (Canada, India, Jordan, New Zealand, Philippines, Tanzania, England and United States), using the hypothesis that a simple checklist, consisting of only 19 items, could improve communication between the teams and the consistency of care in the surgical environment, thereby reducing the numbers of complications and deaths.^8^ The results among 3,733 patients who had undergone operations before the checklist came into use were compared with those among 3,955 patients whose operations took place after its use had started. Use of the list was found to have reduced the risks of death, infection of the surgical site and reoperation.^9^ Indeed: an instrument that took two minute to apply decreased the complication rate by 35% and the mortality rate by 47%.

Meta-analyses have confirmed the importance of sharing information for ensuring that the team’s performance reaches effectiveness^10^ and have shown that effective communication becomes the key to fundamental process such as coordination, cooperation, cognition and conflict resolution.^11^ To facilitate adherence to the WHO checklist, it has been implemented in several counties and institutions around the world and has been adapted to different surgical specialties. There was a clear need to adapt it for use in relation to cardiac and thoracic surgery^12^ in order to attend to critical points that are inherent to these specialties, such as prevention of blood loss, inclusion of extracorporeal circulation and details of patient monitoring and management during transportation to the intensive care unit.

The main cardiothoracic surgery associations in the United States (Society of Thoracic Surgeons, STS) and Europe (European Association for Cardio-Thoracic Surgery, EACTS) have made adaptations to the WHO checklist, while taking care not to remove any item. Rather, they have added specific details for this specialty, including small modifications for adult, congenital, thoracic and transplantation-related cardiac surgery.^13^ Other features that have been implemented have included two terms used in aeronautics: *briefing*, i.e. important instructions that are passed on to the crew at the outset; and *debriefing*, i.e. a report on the mission after the tasks have been executed.^14^ Thus, use of a checklist within cardiothoracic surgery is rated at recommendation level I with evidence level B.

In Brazil, the Ministry of Health has instituted the National Program for Patient Safety (ordinance no. 529/2013), with the aim of contributing to qualification of care in all healthcare services in this country.^15^ Resolution no. 36 of the National Agency for Sanitary Surveillance (Agência Nacional de Vigilância Sanitária, ANVISA), of July 25, 2013, strengthens this program through instituting mandatory actions for promoting patient safety and improving the quality of care.^16^ Among the actions that this legislation establishes, creation of a specific protocol for safe surgery can be cited.^17^ This was drawn up by the Ministry of Health on the basis of the WHO manual “Safe surgery saves lives”.

The Heart Institute (Instituto do Coração, InCor) of Hospital das Clínicas, University of São Paulo Medical School (Faculdade de Medicina da Universidade de São Paulo) implemented its checklist (InCor Checklist) in 2014. It sets forth five steps to be taken for safe surgery: *briefing, sign in, time out, sign out* and *debriefing*. The InCor Checklist started to be applied in 2015 and since 2018 has been used in 100% of cardiothoracic operations at this institution.^18^ The project to implement this checklist included an educational program composed of standardized classes, teaching material, videos and simulations in scenarios that were set up in the surgical center. Surprisingly, an analysis conducted after five years of use of the InCor Checklist showed that this use was associated with a decrease of 58% in surgical mortality at this institution.^19^

One point that we must emphasize is that standardization of the surgical process should not be limited to the surgical center itself, given that several studies have demonstrated that the majority of errors or adverse events (53% to 70%) occur outside of the surgical room, either before or after the operation.^20^ To address this matter, the Surgical Patient Safety System (SURPASS) collaborative group was created.^21^ After implementation of this broader and more systemic checklist, the number of complications diminished from 27.3% to 16.7%, the number of reoperations from 2.7% to 1.1% and the hospital mortality rate from 1.5% to 0.8%.

Thus, we have seen that checklists have had an impact as a central part of surgical safety culture. They have taught us that we all work within a system. Checklists have made specialists better, even with the increasing complexity of their tasks, through the requirement for pauses for verification and checking. Although implementation of checklists is still not a rule within surgery, their use forces us to face up to the fact that we were not in a system. Their use also obliges us to embrace values such as humility, discipline and teamworking, which differ from those through which medicine was created, instead of the values of independence, self-sufficiency and autonomy.

It is time to rethink and understand that we must work within a system. This is the great task of the new generation of healthcare professionals. In every field of medicine, knowledge has been increasing and has been bringing complexity and specialization. No matter how individualistic we might wish to be, complexity requires checklists and teamworking because this is a central part of surgical safety culture.
